# A System for Mixed-Reality Holographic Overlays of Real-Time Rendered 3D-Reconstructed Imaging Using a Video Pass-through Head-Mounted Display—A Pathway to Future Navigation in Chest Wall Surgery

**DOI:** 10.3390/jcm13072080

**Published:** 2024-04-03

**Authors:** Jan Arensmeyer, Benedetta Bedetti, Philipp Schnorr, Jens Buermann, Donatas Zalepugas, Joachim Schmidt, Philipp Feodorovici

**Affiliations:** 1 Division of Thoracic Surgery, Department of General, Thoracic and Vascular Surgery, University Hospital Bonn, 53127 Bonn, Germanyphilipp.feodorovici@ukbonn.de (P.F.); 2 Bonn Surgical Technology Center (BOSTER), University Hospital Bonn, 53227 Bonn, Germany; 3 Department of Thoracic Surgery, Helios Hospital Bonn/Rhein-Sieg, 53123 Bonn, Germany

**Keywords:** mixed reality, augmented reality, thoracic surgery, navigation, surgical planning, image reconstruction, hologram, head-mounted display, volume rendering, image-guided surgery

## Abstract

**Background:** Three-dimensional reconstructions of state-of-the-art high-resolution imaging are progressively being used more for preprocedural assessment in thoracic surgery. It is a promising tool that aims to improve patient-specific treatment planning, for example, for minimally invasive or robotic-assisted lung resections. Increasingly available mixed-reality hardware based on video pass-through technology enables the projection of image data as a hologram onto the patient. We describe the novel method of real-time 3D surgical planning in a mixed-reality setting by presenting three representative cases utilizing volume rendering. **Materials**: A mixed-reality system was set up using a high-performance workstation running a video pass-through-based head-mounted display. Image data from computer tomography were imported and volume-rendered in real-time to be customized through live editing. The image-based hologram was projected onto the patient, highlighting the regions of interest. **Results**: Three oncological cases were selected to explore the potentials of the mixed-reality system. Two of them presented large tumor masses in the thoracic cavity, while a third case presented an unclear lesion of the chest wall. We aligned real-time rendered 3D holographic image data onto the patient allowing us to investigate the relationship between anatomical structures and their respective body position. **Conclusions**: The exploration of holographic overlay has proven to be promising in improving preprocedural surgical planning, particularly for complex oncological tasks in the thoracic surgical field. Further studies on outcome-related surgical planning and navigation should therefore be conducted. Ongoing technological progress of extended reality hardware and intelligent software features will most likely enhance applicability and the range of use in surgical fields within the near future.

## 1. Introduction

High-resolution imaging of thoracic pathologies is widely used, mainly for diagnostic standard of care. Even though powerful applications in computer graphics and the emerging opportunities of the metaverse are increasingly available, the use of 3D-reconstructed imaging is so far only rarely used for surgical planning in thoracic surgery in everyday practice [1,2]. Other approaches to display preoperative 3D patient data in thoracic surgery have been utilizing 3D-printed models [3,4,5]. In a variety of surgical fields, preoperative imaging is progressively displayed in immersive virtual reality (VR) and may be promising to improve accuracy in patient-specific treatment planning [4,6,7,8,9]. In fact, in a recent publication, Bakhuis et al. [10] showed the major impact of 3D-reconstructed imaging inside a virtual reality environment on decision making in lung segmentectomies because a clear anatomic orientation can be not only challenging but also crucial especially for minimally invasive approaches. It has very recently been demonstrated that preoperative 3D surgical planning in VR for oncologic lung segmentectomies can directly add value when integrated into the intraoperative visualization at a surgeon’s console during robotic-assisted thoracoscopy (RATS) [11].

In chest wall-related surgery, technological approaches for 3D surgical planning have been shown [12] and the impact on surgical perception during VR planning has been demonstrated [13]. Unlike in pure lung surgery, chest wall-related procedures frequently face reconstruction challenges. Reconstruction following the removal of chest wall malignancies and infiltrating lung neoplasms may necessitate the utilization of mesh grafts or metal osteosynthesis [14]. In complicated oncological cases, the determination of the treatment strategy involves balancing between sufficient radicality and the restoration of chest wall stability [15,16]. Specifically, for larger defect reconstruction, surgical approaches are discussed in a highly individualized, less-standardized manner and often require a multidisciplinary approach.

### 1.1. Background—Navigation Techniques in Thoracic Surgery

A pioneering role in image-guided navigation has been mainly achieved in the field of neurosurgery. Due to the possibilities of a rigid fixation of the skull using a skull clamp, e.g., Integra LifeSciences Mayfield^®^ (Integra LifeSciences, Princeton, NJ, USA) and the limited mobility of the relatively stable brain, the technological barriers to navigation are not as significant as in extracranial surgery.

In thoracic surgery, a preoperative marking of a lesion is commonly achieved using CT-guided marking with dyes and metallic anchors (hook wires) before the procedure. These invasive methods carry risks of complications such as pneumothorax, bleeding, and wire dislocation as well as additional radiation exposure during the procedure. To minimize morbidity and optimize logistics, these procedures are carried out in some centers immediately prior to surgical treatment or in hybrid operating room settings [17]. In other approaches, the marking of nodules is no longer guided by CT but through bronchoscopy-assisted tagging.

There are solutions that utilize bronchoscopy navigation based on preoperative CT imaging to guide the procedure, where real-time three-dimensional mapping and visualization are applied to navigate a robotic catheter to the target [18]. However, the verification of the lesion’s location still requires the use of ionizing radiation and the corresponding logistical effort.

### 1.2. Background—XR Technology for Image-Guided Surgery

XR, or extended reality, is a term that refers to a group of technologies. The main components of XR are virtual reality (VR), augmented reality (AR), and mixed reality (MR). Whereas virtual reality creates a fully immersive digital environment that simulates the real world or an imaginary one [19], mixed reality creates an environment that blends virtual objects into the physical surrounding to be interacted by the user.

The latest technological progress has led to a wider availability of mixed-reality (MR) hardware including video pass-through technology [20]. It refers to a type of head-mounted display (HMD) that uses cameras to capture the surrounding environment and displays it in real-time on the HMD screens, allowing the user to see both the virtual and real world simultaneously. This opens a wide field of applications in patient care, as it is possible to use these devices in the proximity of the patients. In fact, the fusion of previously acquired imaging with the patient is very well suited for planning and performing a surgical procedure. The 3D data are displayed in a hologram and projected onto the patient. It improves the surgeon’s ability to assess and understand the relationship between the anatomical structures and their respective position in the patient’s body and the target pathology, particularly in cancer treatment [21]. Thus, this on-body projection helps the surgeon understand the relation of anatomical dimensions and improves his ability to choose the intervention method and approach that is required.

So far, previous approaches to image-guided surgical navigation in immersive AR environments have primarily relied on traditional 3D computer graphics that utilize polygonal models, which are typically limited to surface representation and, as a result, appear static to the user. These models are generated from preoperative CT or MRI datasets and subsequently processed into anatomical layers and structures, such as organ vascular trees [22,23,24,25]. However, similar to the characteristics of 3D-printed models, pre-segmented data lack much of the original image information. To address these limitations, real-time volume rendering can be employed, enabling the display of all tissue information from a complete CT scan [26]. In volume rendering, objects or data are represented as a 3D voxel grid in which each voxel contains information about properties such as density or opacity. This technique, however, necessitates significant graphic processing performance and graphic memory on the computer system’s graphic processing unit (GPU) [27,28].

### 1.3. Objective

We describe the novel method of live 3D surgical planning in an MR setting using an HMD with video-pass-through technology and a workstation-based real-time rendering of CT image data. For a better understanding, we describe the method by presenting three representative oncological cases from our institution.

## 2. Materials and Methods

We selected three oncological cases treated in our thoracic surgery department in 2022 and early 2023 for a proof of concept of the technique. Informed consent was obtained from all participants before investigation. The investigation was conducted in accordance with the Declaration of Helsinki. Ethical approval was given by the local ethics committee of the Medical Faculty, Bonn University Hospital, Germany (No. 436/22-EP). The protocols for the individuals’ treatment remained unchanged. Study results were collected independently of clinical assessments and treatment planning. Furthermore, no interventions were performed, and outcome parameters were not evaluated.

The XR system relied on a workstation equipped with one Intel 9900k CPU (Intel Corporation, Santa Clara, CA, USA), 32GB of RAM, and one NVIDIA RTX 3090 graphic processing unit (NVIDIA Corporation, Santa Clara, CA, USA). The head-mounted device (HMD) was a Varjo Technologies Varjo XR-3 (Varjo Technologies Oy, Helsinki, Finland) wired to the workstation using a USB-C cable pair and the Varjo interfaces. Interaction was implemented through a pair of HTC VIVE controllers, (HTC Corporation, Taoyuan, Taiwan), while four SteamVR (HTC Corporation, Taoyuan, Taiwan) base stations 2.0 were used for position tracking. Base stations were placed in the upper room corners ensuring maximum coverage from any location inside the examination room. The used VR DICOM Viewer was Medical Imaging XR, version 0.9.9, and version 0.10.0 (Medicalholodeck AG, Zurich, Switzerland) that had been modified to work with Varjo XR-3, enabling the HMD’s specific features. The latter version underwent implementation of LiDAR capabilities for depth mapping.

**Figure 1:**
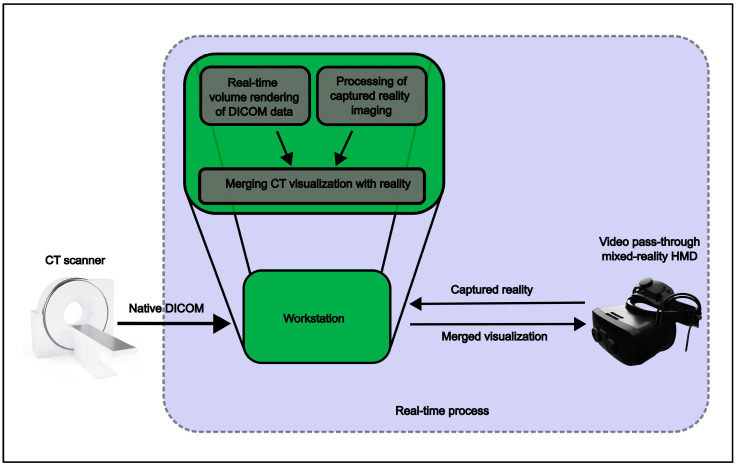
Flowchart on image data and visualization processing.

Image data were collected using a contrast-enhanced computer tomography (CT) scan with a slice thickness equal to or smaller 1 mm and saved as DICOM. The datasets were transferred into the system using a USB data carrier. After importing DICOM data into the application, the slices were transformed into a 3D pixel cloud. Figure 1 illustrates the data flow of the system, highlighting the real-time process of workstation-based volume rendering and incorporating the pass-through stream. Visualization of the reconstructed CT image data was adapted through tissue windowing, color grading, and cropping to expose the region of interest (e.g., tumor mass and/or vascular system). Hounsfield coupled windowing and color grading were variably adjusted by visual feedback of the displayed dataset. Adjusted visualization was saved as a reproducible preset. All examiners were previously trained in operating the controllers, general software functions, and editing tools. Each case was examined by two surgeons, each with a minimum experience of 4 years in the field of thoracic surgery. One surgeon operated the tools in the MR environment, while the other observed and assisted through a 2D stream displayed on the workstation’s monitor.

**Figure 2:**
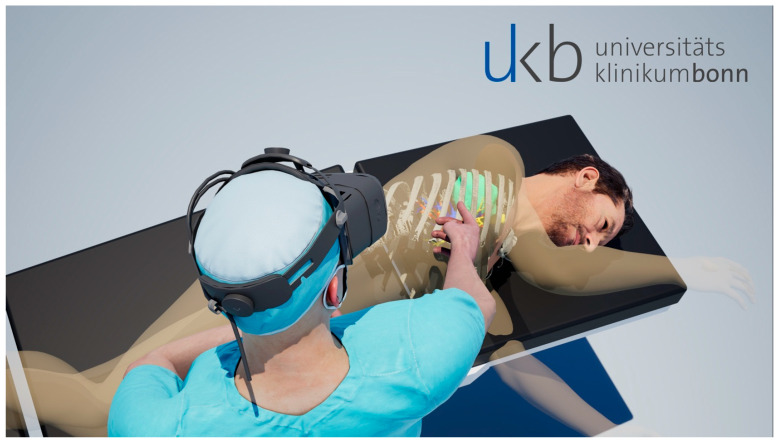
Illustration of mixed-reality examination setup with image overlay.

The patient was placed on an examination table with his upper body exposed. The hologram projected in the room was first windowed by the user, displaying the patient’s skin surface. The hologram was then transferred to the patient’s upper body. Anatomical landmarks were used for position adjustment. Suitable landmarks were the jugulum, both clavicles, the costal arch, and the mammilla in men. Accurate alignment was ensured by visually checking the correspondence of the anatomical landmarks. The software was set to receive the 3D depth map of the HMD’s light detection and ranging (LiDAR) sensor. Through this, any object in the range of 60 cm around the examiner will overrule the virtual displays such as the reconstructed CT scan. This feedback was used to further improve the manual positioning of the CT scan onto the patient’s body. As soon as the correct alignment was implemented, the tissue windowing was reverted to display the area of interest. In addition, a dynamic slicing tool offering three degrees of freedom was applied to expose and highlight areas of interest. Examinations were recorded, capturing the content of both the virtual and real environments simultaneously in an MPEG4 file format for interpretation. Figure 2 illustrates the examination setup in a third-person view, simulating the examiner’s holographic vision of CT data.

## 3. Results

Real-time reconstructed high-resolution thoracic CT scans were used in three oncological cases of male patients, two presenting with large tumor masses inside the chest and one presenting with a potentially malignant lesion of the 7th rib of the right dorsal hemithorax. There were no oncologic conditions in the previous history of all three patients. A landmark-adjusted overlay was initiated by windowing the reconstructed pixel cloud to display the patient’s skin surface, as can be seen in Figure 1. The regions of interest were then pointed out using the integrated tools for tissue filtering, color grading, and opacity adjustments.

**Figure 3:**
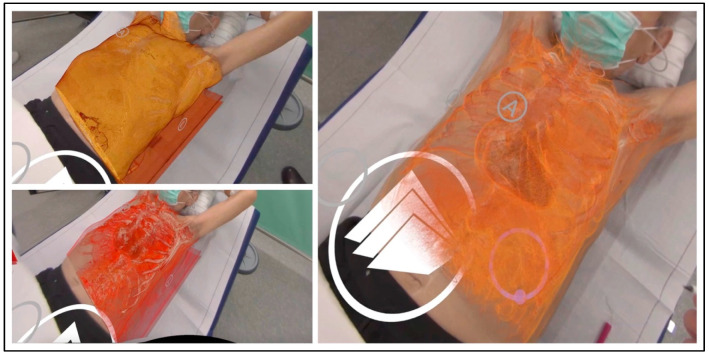
Alignment and windowing of high-resolution reconstructed image on patient with large epithelioid sarcoma. The anterior side of the rendered CT image is labeled Ⓐ.

The first patient presented a large mass of an epithelioid sarcoma located in the upper part of the left hemithorax. The tumor showed broad contact with the mediastinal structures, which were slightly shifted to the contralateral side. The left lung was exceedingly compressed toward the dorsal chest wall. Figure 3 presents the dynamic tissue windowing and color grading during the XR examination, adjusting the intensity of skin, soft tissue, costal, and tumor visualization to comprehend the surgical assessment.

**Figure 4:**
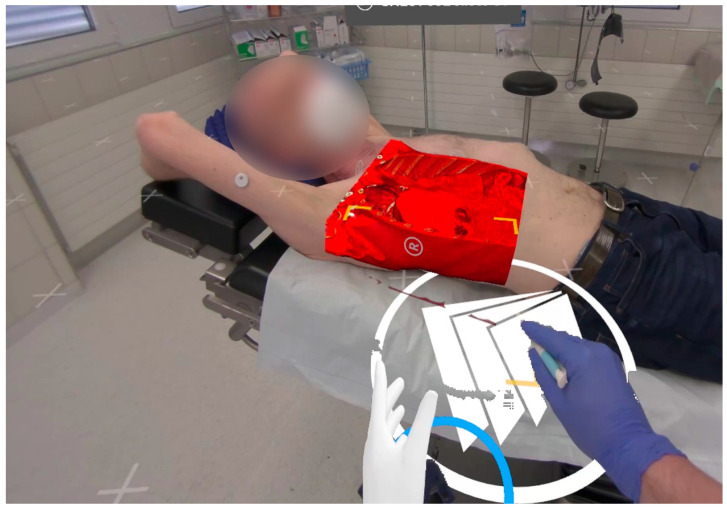
Slicing through overlayed 3D image to examine tumor mass of a solitary fibrous tumor in the right thorax. The right side of the rendered CT image is labeled Ⓡ.

The second patient presented with a solitary fibrous tumor (SFT) of the right pleura with a Doege-Potter Syndrome compressing the upper and middle lobe of the right lung. The integrated slicing tool was utilized to assess potential tumor infiltration in the chest wall, diaphragm, and central structures, as depicted in Figure 4.

**Figure 5:**
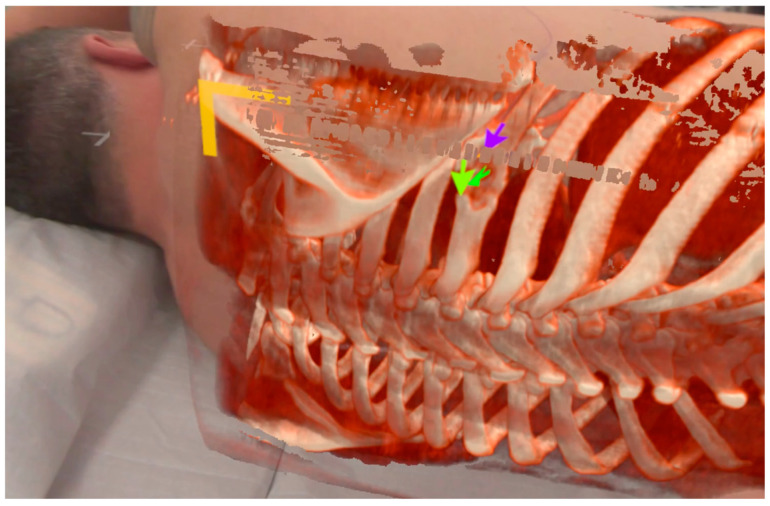
Overlayed rib cage with virtual markings of a costal lesion. The proximal ridge (green arrows) and the distal ridge (purple arrow) have been labeled during case assessment.

A third patient was diagnosed with an unclear suspected malignant lesion of the 7th rib of the right dorsal thorax with central osteolysis. During the XR assessment, the target was highlighted as displayed in Figure 5. The rib lesion turned out to be benign in the histopathological findings after the partial rib resection was performed. It was most likely caused by an unrecognized fracture from the past with excessive callus formation.

In all cases, image overlay could be performed while displaying the skin surface of the dataset. Examination times were 25 min in the first case, 23 min in the second case, and 27 min in the third case. No malfunctions were reported by the examiners. By utilizing the real-time tools for image manipulation, the target regions were sufficiently identified and highlighted by the examiner. In addition, the examiners reported that key structures of soft and hard tissues, such as muscular layers of the dorsal chest wall, main mediastinal vessels, and sternal boundaries, could be distinguished, and their geometric relationship to the target lesion could be determined. For this purpose, additional tools such as fixed slices, arrow annotations, or freehand 3D drawing were utilized, as shown in the figures. Furthermore, measurements were conducted within the MR software (Medical Imaging XR, version 0.9.9, and version 0.10.0, Medicalholodeck AG, Zurich, Switzerland) to determine distances, extents, and potential defect areas.

During the examination of the first patient, a troubling issue was observed: depth perception became impeded due to the absence of superimposition of closer objects over more distant digital content. This issue particularly impacted perception during manual interaction with the patient. To ameliorate this, the adapted software version 0.10.0 was used for patients 2 and 3. The software utilized LiDAR sensor technology integrated within the Varjo XR-3. It was configured to overwrite digital content with a depth map of the environment within a 60 cm distance from the examiner’s position. The effect of this modification is clearly discernible in the ‘cutout’ of the examiner’s hand, as depicted in Figure 5. LIDAR-based surface mapping caused the overwriting of the patient’s surface onto the surface of the holographic overlay. This created a phenomenon characterized by artifacts resembling ‘freckles’ upon the matching of the surfaces (within the range of 60 cm from the examiner). This distinctive occurrence proved to be helpful as an indicative marker for a good overlay positioning. It should be emphasized that the alignment was meticulously readjusted in every case until this phenomenon was consistently observed.

Furthermore, it should be noted that alterations in the patient’s positioning relative to the initial CT scan led to aberrations in the representation of the holographic overlay on the patient’s body. For instance, in the third case, the patient’s lateral position allowed gravitational forces to compress the left hemithorax, resulting in a deformity that was not accurately reflected in the holographic overlay. To mitigate the extent of this deviation concerning the displayed pathology (i.e., the tumor), reference points in close anatomical proximity were utilized to align the holographic overlay.

After proper alignment, potential surgical approaches and the best possibilities to access the target areas in the least invasive way were discussed.

## 4. Discussion

To the best of our knowledge, we report the first-time utilization of real-time volume-rendered CT imaging as a holographic overlay in an MR pass-through environment for navigational approaches in chest wall-related surgery. As a key advantage, intuitive perception through 3D on-body visualization to determine the location of a small costal [rib] lesion may be highly beneficial when utilized in a future real-time application during procedures, minimizing intraoperative radiation-based guidance. Moreover, we demonstrated a comprehensive approach to assessing complex conditions of the thoracic cavity while analyzing cases with large tumor masses. Hence, our investigation suggests true benefits of a 3D visualization of the tumors’ dimensions to distinguish the open surgical approach, such as the necessity of a sternotomy or the extent of a lateral thoracotomy. During our investigation, several factors influenced the examination time. Both a careful surgical review and technical readjustments were relevant. Furthermore, the novelty of the method to the examiners led to prolonged user interaction.

Video pass-through technology appears promising in addressing the limitations of optical see-through devices, which still have a prominent position on the market of commercially available HMDs [29,30]. The hardware currently available in this segment is often expensive to acquire. Operating these systems requires suitable facilities, as well as advanced expertise in calibrating and adjusting XR systems. Additionally, a workstation-based setup with a tethered HMD limits mobility and flexibility in accommodating other use cases. It should be emphasized that pass-through technology faces significant regulatory hurdles if it is intended for intraoperative use and may not be approved by federal agencies.

Currently released HMDs are equipped with video pass-through technology and improved on-board performance. They are expected to serve a wide range of consumer and enterprise use cases in the near future, given the intense promotion of spatial computing development [31]. Therefore, affordable hardware will most likely amplify the expansion of mixed-reality applications in surgical science. While computing performance in stand-alone HMDs remains a limiting issue for complex rendering tasks, cloud-based solutions for data storage and rendering tasks might serve well to scale up research and development in healthcare facilities, requiring solid connectivity through broadband networks such as 5G [32].

While surface visualization using polygon meshes may suffice for certain inquiries, such as assessing the topographical relationships of individual anatomical structures, and requires moderate computing power, interactive volume rendering, which demonstrates all anatomical layers analogous to the surgery itself, appears to be essential for 3D treatment planning and navigation. The dynamic workflow in data visualization, which involves applying tissue windowing, color grading, and opacity settings, enables the surgeon to gather the required information about the entire surgical site within minutes. This benefit of being able to rapidly adjust the visualization between tissue entities seems to be particularly relevant for chest wall procedures, where surgeons must incorporate considerations for soft tissue, muscle, and costal conditions for both resection and defect reconstruction. However, the process of manual mapping during the alignment of image overlay suggests potential limitations in accuracy, which may significantly depend on the examiner’s experience.

Continuing developments in computer graphics demonstrate advanced approaches for representing volumetric data, aiming to provide a more photo-realistic representation of 3D data [33]. This illumination technique enhances the natural appearance of a model, potentially leading to a more accurate surgical assessment, even in VR environments [34]. Additionally, intelligent image processing software, which offers autonomous segmentation and classification, is increasingly being implemented in mixed-reality environments. This technology could prove highly beneficial for the development of image-guided visual navigation in a broad spectrum of surgical fields [35]. These AI-based classifiers enable users to interactively select targets of interests, such as lung lobes or even lung segments, individual ribs, and vertebrae. This automatically generated selective visualization streamlines manual data processing and may serve to provide a more efficient and precise preoperative surgical assessment. It is also conceivable that solutions for the automated overlay of imaging through previously trained AI-based registration models will also be introduced into the application in the near future. In robotic-assisted procedures, recently published AI models in the field of surgical computer vision may contribute to realizing navigational imaging overlays through real-time de-occlusion of surgical instruments [36].

As with other innovations, the widespread adoption of technology depends heavily on usability. The future design of user interfaces (UIs) might be the crucial step for the overall acceptance of XR and is most likely essential for surgical use cases in sterile environments. Therefore, intuitive UIs, such as the capture of hand gestures through hand tracking and the elimination of hand-held controllers, contribute to improving user experience, despite advantages not yet being proven in medical applications [37,38]. In addition to advanced hand tracking, both current and future voice control models, as well as eye tracking combined with hand gestures, could effectively serve for user interaction in an XR environment [39]. Looking ahead, the potential future integration of generative artificial intelligence (AI) suggests that virtual assistants could serve not only as sources of knowledge but also for 3D modeling tasks in surgical applications using XR [40].

## 5. Conclusions

Thoracic chest wall surgery—especially in a minimally invasive setting—includes the question of the best approach to the target area. This investigation into the holographic overlay of real-time 3D-reconstructed imaging through volume rendering in an extended reality environment, utilizing a video pass-through system on three complex surgical cases, shows promise as an advanced tool for preprocedural surgical planning. Previewing spatial relations through a first-person surgical perspective, in contrast to traditional preoperative preparation using conventional 2D greyscale image slices, could become significantly important in planning extensive, less-standardized thoracic operations. The features of the real-time volume-rendering technique allow for continuous interaction and manipulation of the data to efficiently obtain and visualize the relevant information for the surgeon. More specifically, preoperative assessment in mixed reality could greatly benefit chest wall-related tasks. Surgical targets in this region are embedded in a relatively static environment compared to lung parenchyma but are often challenging to localize and necessitate accurate resection margins, such as costal lesions [41]. In this investigation, we examined patients with larger or prominent tumors to determine the potential surgical strategy. The relevant benefit of XR technologies will be in approaching smaller lesions during minimally invasive and robotic-assisted procedures, providing intraoperative decision support and guidance, especially considering the lack of haptic detection of smaller lesions.

Additional studies will be conducted to evaluate the feasibility of this novel method for approach and resection planning in both complex oncological and non-oncological cases. These studies aim to gather technical data on accuracy and, furthermore, investigate clinical outcomes. The technological pathway to continuous image-guided navigation based on preoperative data will have to address aspects of advanced surface registration and tissue deformation.

## Data Availability

The data presented in this study are available on request from the corresponding author. The data are not publicly available due to data privacy regulations.

## Abbreviations

3D	Three-Dimensional
5G	Fifth-Generation Technology Standard for Broadband Cellular Networks
CPU	Central Processing Unit
CT	Computed Tomography
DICOM	Digital Imaging and Communications in Medicine (Platform)
GPU	Graphic Processing Unit
HMD	Head-Mounted Display
MR	Mixed Reality
RAM	Random-Access Memory
RATS	Robotic-assisted Thoracoscopy
SFT	Solitary Fibrous Tumor
UI	User Interface
VR	Virtual Reality
XR	Extended Reality
